# THE ROLE OF GEOGRAPHIC-LEVEL RACE AND SOCIOECONOMIC FACTORS ON ACCESS TO HIGH QUALITY MEDICARE HOME HEALTH

**DOI:** 10.1093/geroni/igz038.2869

**Published:** 2019-11-08

**Authors:** Shekinah A Fashaw, Kali Thomas

**Affiliations:** Brown University, Providence, Rhode Island, United States

## Abstract

Prior research suggests that neighborhoods with predominantly minority and low-income individuals have decreased access to high quality hospitals, primary care physicians, and nursing homes. The purpose of this study is to determine if low quality Home Health Agencies (HHAs) are concentrated within more disadvantaged neighborhoods, while high quality HHAs cluster in more affluent neighborhoods. We characterize neighborhoods by racial and ethnic composition, and the proportion of residents below the federal poverty line; and HHAs according to their star ratings. We conduct a national, observational descriptive study using data from the 2017 HH Compare and American Community Survey. Predominantly black neighborhoods are served by the highest proportion of unrated and low-rated HHAs, while predominantly white neighborhoods have the highest proportion of high quality HHAs. This study signals and potential explanation for why minorities and duals receive care from lower quality HHAs. Such knowledge may help inform reimbursement and incentive practices.

